# The Potential of Angiogenin as a Serum Biomarker for Diseases: Systematic Review and Meta-Analysis

**DOI:** 10.1155/2018/1984718

**Published:** 2018-03-15

**Authors:** Dongdong Yu, Yikai Cai, Wei Zhou, Jinghao Sheng, Zhengping Xu

**Affiliations:** ^1^Institute of Environmental Health, School of Public Health, Zhejiang University, Hangzhou 310058, China; ^2^Collaborative Innovation Center for Diagnosis and Treatment of Infectious Diseases, Zhejiang University, Hangzhou 310058, China; ^3^Program in Molecular and Cellular Biology, Zhejiang University, Hangzhou 310058, China; ^4^Department of General Surgery, Sir Run Run Shaw Hospital, School of Medicine, Zhejiang University, Hangzhou 310058, China

## Abstract

**Background:**

Angiogenin (ANG) is a multifunctional angiogenic protein that participates in both normal development and diseases. Abnormal serum ANG levels are commonly reported in various diseases. However, whether ANG can serve as a diagnostic or prognostic marker for different diseases remains a matter of debate.

**Methods:**

Here, we performed a systematic review and meta-analysis of the literature utilizing PubMed, Web of Science, and Scopus search engines to identify all publications comparing plasma or serum ANG levels between patients with different diseases and healthy controls, as were studies evaluating circulating ANG levels in healthy populations, pregnant women, or other demographic populations.

**Results:**

This study demonstrated that the serum ANG concentration in healthy populations was 336.14 ± 142.83 ng/ml and remained relatively stable in different populations and regions. We noted no significant differences in serum ANG levels between patients and healthy controls, except in cases in which patients suffered from cancer or cardiovascular diseases. The serum ANG concentrations were significantly higher in patients who developed colorectal cancer, acute myeloid leukemia, multiple myeloma, myelodysplastic syndromes, and heart failure than those in healthy controls.

**Conclusion:**

ANG has the potential of being a serum biomarker for cancers and cardiovascular diseases.

## 1. Introduction

Angiogenin (ANG), the fifth member of the vertebrate-specific secreted ribonuclease A superfamily, is a multifunctional proangiogenic protein comprising a signal peptide whose cleavage facilitates its secretion. ANG is a potent inducer of blood vessel formation, which plays roles in several physiological and pathological processes, including tumorigenesis [1], neuroprotection [2], inflammation [3], host defense [4], reproduction [5], wound healing [6], and hematopoietic regeneration [7]. To induce angiogenesis, secreted ANG must bind to the membrane surface actin of vessel endothelial cells to activate the matrix protease cascades, thus degrading the basement membrane and extracellular matrix to allow endothelial cell to penetrate and migrate. The sparse endothelial cells then express ANG receptors which mediate ANG nuclear translocation to enhance ribosome biogenesis, resulting in endothelial cell proliferation. The proliferated endothelial cells eventually form new blood tube and are deposited by smooth muscle cells, which also are stimulated by ANG, for maturation as a new blood vessel [8]. Since ANG plays key roles in cell growth and survival, abnormal ANG levels or mutations are commonly observed in a variety of diseases, suggesting that ANG may be a valuable diagnostic or prognostic marker for certain diseases.

The presence of ANG in normal plasma or serum, wherein its concentration is estimated to range from 60 to 150 ng/ml, was initially noted in 1987 [9]. A highly sensitive immunoenzymometric assay capable of detecting ANG was developed in 1993. This assay showed that serum ANG concentrations ranged from 111 to 380 ng/ml [10]. Researchers have since used enzyme-linked immunosorbent assay (ELISA) to measure serum ANG levels in different physiological and pathological conditions. Many studies have investigated serum ANG levels in healthy people and patients with different diseases; however, these studies obtained conflicting results.

For example, Montero et al. reported that serum ANG concentrations were significantly elevated in patients with breast cancer [11]; however, others did not observe this phenomenon [12–14]. Two of three studies investigating the association between serum ANG levels and amyotrophic lateral sclerosis (ALS) reported that serum ANG levels were elevated in affected patients compared with those in control subjects [15, 16]; however, the other study drew the opposite conclusion [17]. These inconsistent results may be attributable to the limited numbers of subjects, as well as to small differences between the patients and healthy controls enrolled in each study. A systematic review may be able to highlight the problems affecting the results of individual studies and determine whether future research regarding specific issues is needed [18].

In this study, we conducted a comprehensive systematic review and meta-analysis of published studies on the association between serum ANG levels and diseases to explore whether serum ANG has the potential to serve as a diagnostic marker for certain diseases.

## 2. Results

### 2.1. Literature Search Results and Quality Assessment of the Literature Included in the Analysis

**Figure 1:**
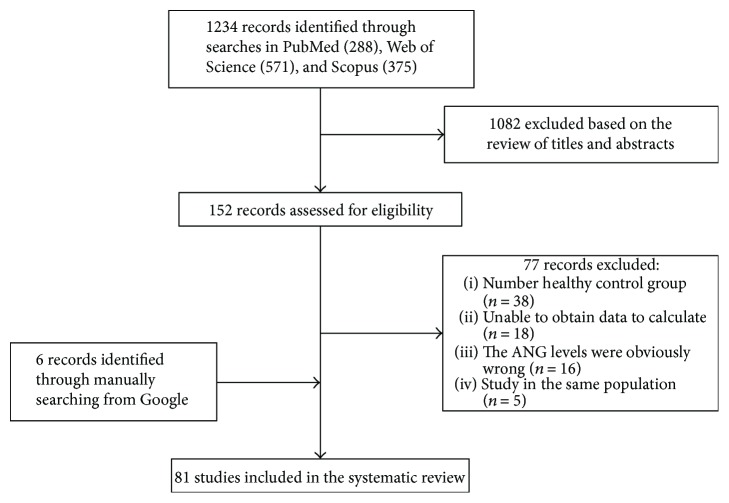
Article selection process. The flow diagram of the systematic literature search and its result, as well as the data regarding the specific studies identified via specific database searches, the numbers of hits, and the reasons for study exclusion.

As demonstrated in Figure 1, a total of 1234 potentially relevant studies were retrieved from three databases, and 152 of these studies were considered relevant based on their titles and abstracts. Of the 152 studies, 77 were excluded from the analysis because they did not meet the above inclusion criteria. Specifically, 38 studies lacked a healthy control group, 18 studies lacked sufficient data for analysis, 16 studies reported incorrect data pertaining to serum ANG levels, and 5 studies used the same population. We also manually screened 6 studies identified via Google searches. Ultimately, 81 articles fulfilled the above inclusion criteria and were included in the analysis. Of these studies, 37 reported on cancers, 8 reported on diseases of the cardiovascular system, 5 reported on neurodegenerative diseases, 8 reported on diabetes, 7 reported on abnormal pregnancies, 9 reported on other diseases, and 7 reported on healthy populations. The main characteristics of these studies are summarized in Supplementary Table 1. The results of the quality assessment, which was conducted using the NOS, are also shown in Supplementary Table 1 (the quality assessment involved only studies eligible for the meta-analysis; 63 studies were included therein). The scores of the studies included in the analysis ranged from 5–8 points, and 65% (41/63) of the eligible studies received scores of ≥7 points, indicating that they were high-quality studies.

### 2.2. Serum ANG Levels in Healthy Populations

Although the serum ANG level in a healthy population ranges from approximately 200 to 300 ng/ml, according to published data, there is no general consensus regarding a reference value for this population. Moreover, the associations between serum ANG levels and patient demographics (region, age, and gender) remain unclear. Thus, we pooled and analyzed the serum ANG levels of healthy control subjects from 62 studies (7 studies on adverse pregnancy and 1 study on the perinatal period were excluded because they included data pertaining to special patient demographic characteristics, and 11 studies were excluded because they did not meet the inclusion criteria pertaining to the collection of study data that measured ANG levels with the same method (ELISA kit from R&D Systems Inc.)). A total of 3876 healthy controls with an average level of 336.14 ng/ml (standard deviation 142.83 ng/ml), a level slightly higher than the currently recognized average, were included in the analysis.

**Figure 2:**
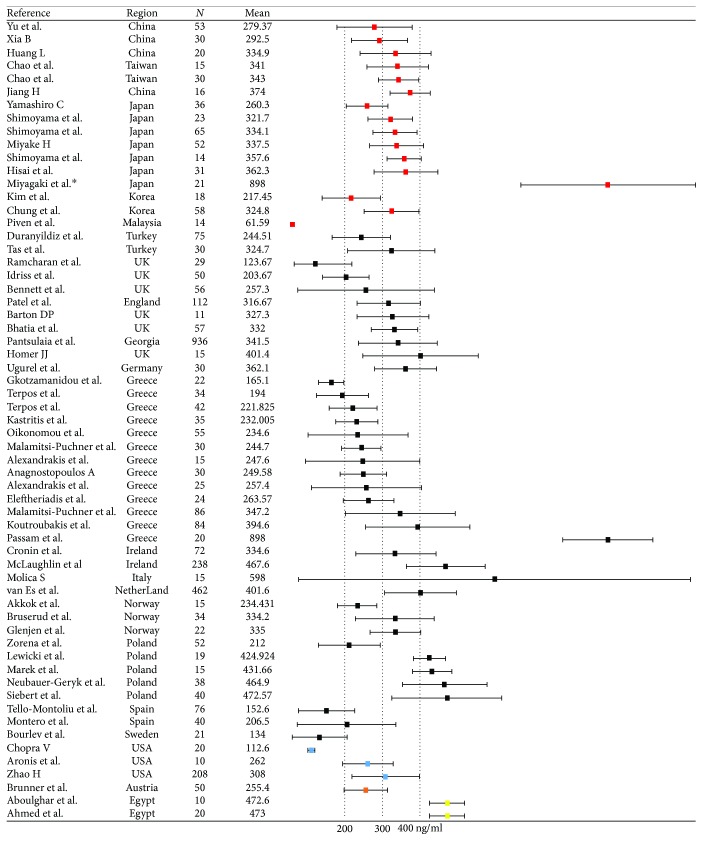
Forest plot demonstrating serum ANG levels in healthy controls. The squares indicate the mean serum ANG level, and the horizontal lines indicate the standard deviation (SD). ^∗^Two studies used the same healthy control sample, only one study was represented; *N*: number of subjects.

We also compared serum ANG levels between different healthy populations organized according to region, age, and gender (Figure 2). Region analysis showed that the average serum ANG levels in subpopulations comprising Asian, European, American, Austrian, and African individuals were approximately 335.52 ± 154.78 ng/ml (*n* = 496, 16 studies), 339.83 ± 143.64 ng/ml (*n* = 3062, 39 studies), 289.65 ± 100.10 ng/ml (*n* = 238, 3 studies), 255.40 ± 56.57 ng/ml (*n* = 50, 1 study), and 472.87 ± 45.07 ng/ml (*n* = 30, 2 studies), respectively. There was no difference in serum ANG levels between the Asian and European populations. No statistical analyses of the data pertaining to the American, African, or Austrian populations were performed because the number of studies assessing these populations was small. These results indicated that serum ANG levels remained fairly stable overall; however, there were individual studies in which this was not the case. For example, Piven et al. reported that serum ANG levels were extremely low in Malaysia [19] and that the Spanish and Swedish populations also had lower ANG levels, as the average value for these populations was 165.49 ng/ml; however, 4/5 studies evaluating these populations showed that the average serum ANG level in the Polish population was higher than 400 ng/ml. In addition, a study aimed to determine whether differences in circulating angiogenesis markers existed among different ethnic/racial groups. This study found that differences in circulating angiogenesis marker levels existed among South Asian, Black African-Caribbean, and Caucasian populations, as serum ANG levels were higher in South Asian and Black African-Caribbean populations than those in Caucasian European populations [20]. We noted no significant difference in serum ANG levels between the Asian and European populations.

Several papers compared serum ANG levels between males and females (Supplementary Table 1). A community-based population study showed that serum ANG levels were significantly higher in men than those in women [21]; however, other studies performed simultaneously reported contrasting and nonsignificant results [13, 22, 23]. Pooled data analysis indicated that there was no significant difference in serum ANG between males and females (*p* = 0.519) (Figure S1). Malamitsi-Puchner et al. analyzed serum ANG levels in patients of different ages and found that they increased significantly from the fetal stage to adulthood [24]. In another study, Bruserud et al. reported that elderly individuals had higher preactivity serum ANG levels than young athletes [25]. These data suggest that serum ANG levels may increase with aging. However, no statistical analyses of the relationship between serum ANG levels and age could be performed because only limited amounts of data pertaining to specific age groups were available. Taken together, these findings indicate that serum ANG levels remain relatively stable in different populations.

### 2.3. Serum ANG Levels in Patients

#### 2.3.1. Cancers

**Figure 3:**
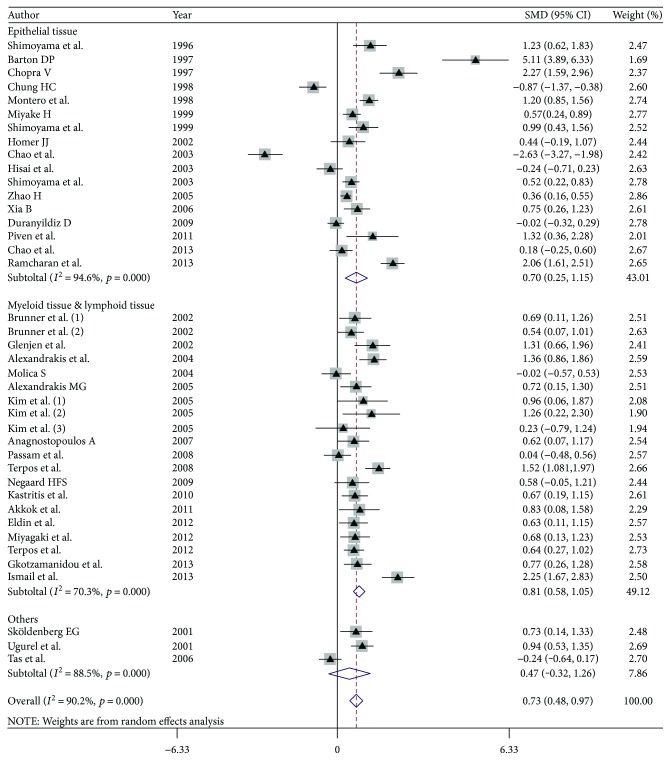
Forest plot comparing serum ANG levels in patients with cancer with those in healthy controls. Image of the subgroup analysis of the studies comparing serum ANG levels in patients with cancers origins from different cancer cells with those in healthy controls. Brunner B (1) and Brunner B (2) from the same study; Brunner B (1) studied AML and Brunner B (2) studied MDS. Kim JG (1), Kim JG (2), and Kim JG (3) from the same study studied AML, CML, and ALL separately.

ANG was originally isolated from cultured tumor cells [26] and was found to have the capacity to enhance primary and metastatic tumor cell growth. ANG levels have been assessed in various types of cancer to establish the relationship between serum ANG levels and cancer progression. Here, we performed a meta-analysis of 37 case-control studies (Figure 3). Our pooled estimates indicated that serum ANG levels in patients with cancer were significantly higher than those in healthy controls (pooled SMD = 0.728, 95% CI = 0.484 to 0.973, *p* < 0.001). Specifically, serum ANG levels in patients with cancer were 96.21 ng/ml (95% CI = 69.85 to 122.57) higher than those in corresponding healthy controls, according to the results of the pooled data analysis. We subsequently divided the studies into the following subgroups (Figure 3), according to the origins of the cancer cells described in each study: an epithelial cell group (*n* = 17 studies) and a myeloid and lymphoid cell group (*n* = 17 studies). No significant difference in serum ANG levels was noted between the subgroups (*p* = 0.12). Regarding serum ANG levels in patients with specific types of cancer, patients with colorectal cancer (CRC) [27, 28] (pooled SMD = 1.542, 95% CI = 0.496 to 2.587, *p* = 0.004), acute myelogenous leukemia (AML) [29–32] (pooled SMD = 1.319, 95% CI = 0.574 to 2.064, *p* = 0.001), multiple myeloma (MM) [33–37] (pooled SMD = 0.822, 95% CI = 0.604 to 1.040, *p* < 0.001), and myelodysplastic syndromes (MDSs) [29, 38] (pooled SMD = 0.616, 95% CI = 0.254 to 0.978, *p* = 0.001) had significantly higher serum ANG levels than healthy controls; however, patients with hepatocellular cancer (HCC) [39–41] (*p* = 0.249), breast cancer [12, 19, 42, 43] (*p* = 0.443), non-Hodgkin lymphomas (NHLs) [44, 45] (*p* = 0.257), and melanoma [46, 47] (*p* = 0.550) did not have significantly higher serum ANG levels than healthy controls (Figure S2). According to these results, we concluded that serum ANG levels are upregulated in patients with cancer, especially patients with CRC, AML, MM, melanoma, and MDSs.

#### 2.3.2. Cardiovascular Diseases

**Figure 4:**
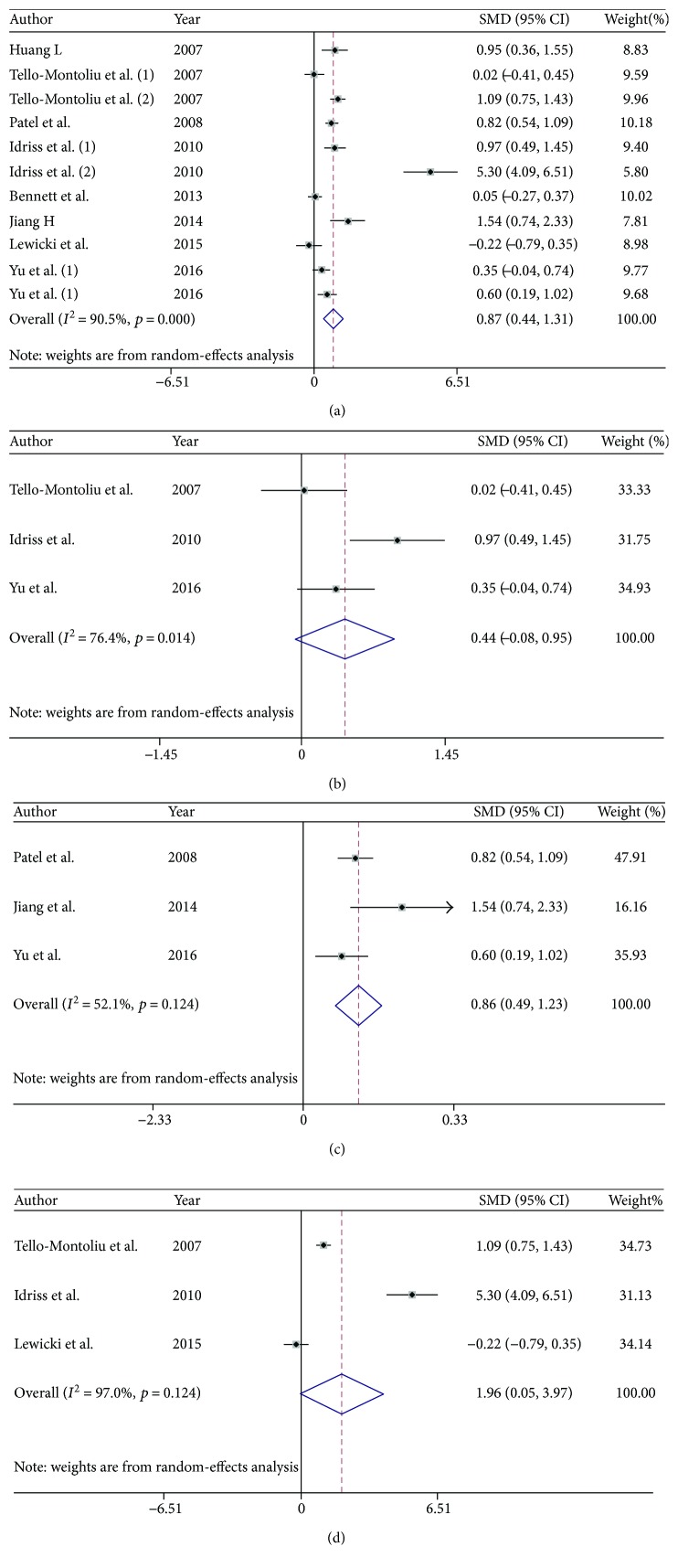
Meta-analysis of studies investigating the relationships between serum ANG levels and cardiovascular diseases. The images of the (a) forest plot comparing serum ANG levels in patients with CVD with those in healthy controls. Tello-Montoliu (1) and Tello-Montoliu (2) from the same study studied CAD and ACS. Idriss NK (1) and Idriss NK (2) from the same study studied CAD and ACS. Yu P (1) and Yu P (2) from the same study studied CAD and heart failure. (b) Forest plot comparing serum ANG levels in patients with CAD with those in healthy controls. (c) Forest plot comparing serum ANG levels in patients with heart failure with those in healthy controls. (d) Forest plot comparing serum ANG levels in patients with ACS with those in healthy controls.

Angiogenesis is common in cardiovascular disease (CVD), which comprises coronary artery disease (CAD), acute coronary syndrome (ACS), heart failure, and other diseases. ANG is a potent inducer of neovascularization; thus, its level in the circulation reflects the degree to which various angiogenic processes, including increases in vessel permeability, endothelial proliferation, and vascular maturation, have occurred. Thus, ANG may be useful as an indicator of vascular disease progression. Here, we performed a meta-analysis of 8 studies investigating the relationships between ANG levels and cardiovascular diseases (Figure 4). Our pooled estimates indicated that serum ANG levels in patients with CVD were significantly higher than those in healthy control subjects (pooled SMD = 0.874, 95% CI = 0.442 to 1.307, *p* < 0.001), as serum ANG levels were 116.22 ng/ml higher in patients with CVD than those in corresponding healthy controls. Regarding ANG levels in specific subtypes of cardiovascular disease, pooled analysis showed that serum ANG levels were elevated in patients with CAD compared with those in healthy controls; however, without significant difference (pooled SMD = 0.435, 95% CI = −0.079 to 0.949, *p* = 0.097) (Figure 4). Pooled analysis also showed that serum ANG levels were not significantly different between patients with ACS and healthy controls (pooled SMD = 1.956, 95% CI = −0.054 to 3.966, *p* = 0.057) [48–50]. Moreover, patients with heart failure, the typical end stage of most CVDs, presented with increased serum ANG levels compared with healthy controls, according to the results of the pooled analysis (pooled SMD = 0.812, 95% CI = 0.592 to 1.032, *p* < 0.001) [51–53]. Taken together, our results show that increased serum ANG levels are related to cardiac functional deterioration.

#### 2.3.3. Neurodegenerative Diseases

Genetic studies have shown that certain ANG mutations are related to neurodegenerative diseases, such as ALS and PD [54, 55]. Other studies have demonstrated that ANG mutations result in protein function loss and that the wild-type ANG protein is a very potent neuroprotective factor [56]. However, it remains unclear whether serum ANG levels are associated with neurodegenerative diseases. We found that 5 studies were eligible for inclusion in the analysis (Supplementary Table 1) and that serum ANG levels were not significantly different between patients with neurodegenerative diseases and healthy controls (pooled SMD = 0.012, 95% CI = − 0.379 to 0.403, *p* = 0.953) (Figure S3). The average serum ANG level in patients with neurodegenerative diseases was approximately 368.80 ± 141.42 ng/ml (*n* = 1026). Regarding ANG levels in specific types of neurodegenerative diseases, serum ANG levels were not significantly different between healthy controls and patients with AD (*p* = 0.685, *n* = 225) [57, 58], ALS (*p* = 0.484, *n* = 638) [15–17], and PD (*p* = 0.72, *n* = 163) [16] (Figure S3). Based on these results, we reasoned that ANG functional and structural integrity is more important than serum ANG levels with respect to the pathogenesis of neurodegenerative diseases.

#### 2.3.4. Diabetes

Diabetes is strongly associated with both microvascular and macrovascular diseases; thus, its pathogenesis may be related to the abnormal expression of proangiogenic factors, such as ANG. There were 8 studies reported on the association between diabetes and serum ANG levels. Four of these studies investigated the associations between serum ANG levels and type 1 diabetes mellitus (T1DM) [59–62], 3 studies investigated the associations between serum ANG levels and type 2 diabetes mellitus (T2DM) [63–65], and 1 study investigated the associations between serum ANG levels and both diseases [66] (Supplementary Table 1). Our pooled data showed that serum ANG levels were higher in patients with T1DM than those in healthy controls; however, the difference between the two groups was not statistically significant (pooled SMD = 0.632, 95% CI = −0.341 to 1.605, *p* = 0.203) (Figure S4). The average serum ANG level in patients with T1DM was approximately 407.52 ± 135.26 ng/ml (*n* = 181). Serum ANG levels were decreased in patients with T2DM compared with those in healthy controls when the data were pooled together, but there was no significant difference in serum ANG levels between the two groups (pooled SMD = −1.455, 95% CI = −3.201 to 0.291, *p* = 0.102). The average serum ANG level in patients with T2DM was approximately 366.18 ± 126.04 ng/ml (*n* = 132). Although the cause of the contrasting results described above is largely unknown, these results reveal that the process by which angiogenesis occurs in T1DM is different than the process by which it occurs in T2DM. Further studies with larger samples are required to better understand this issue.

#### 2.3.5. Other Physiological and Pathological Processes

ANG also plays roles in other physiological and pathological processes. Seven studies regarding ANG levels in pregnancy and 8 studies regarding ANG levels in other diseases (Supplementary Table 1) fulfilled our inclusion criteria. However, a meta-analysis was not conducted because of heterogeneity among the studies with respect to their designs. Thus, we performed only a systematic review of the studies.

A case-control study reported that serum ANG levels increase between the 10th and 40th weeks of pregnancy and that serum ANG levels are correlated with the week of gestation [67]. Serum ANG levels also increased rapidly in full-term pregnancies [68], and ANG levels in the umbilical cord blood were significantly higher in full-term fetuses than those in preterm fetuses [69]. However, maternal and fetal circulating ANG levels were not different between pregnancies with small-for-gestational-age (SGA) fetuses and pregnancies with appropriate-for-gestational-age (AGA) fetuses [70]. Thus, ANG may play an important role in vascular development during pregnancy. A study reported that serum ANG levels were lower in normal pregnancies than those in pregnancies complicated by hypertension [71]. However, we included 4 studies regarding ANG levels in preeclampsia in the meta-analysis [67, 72–74], which showed that there was no significant difference in ANG levels between pregnant women who developed preeclampsia and pregnant women who did not develop preeclampsia (pooled SMD = 0.438, 95% CI = −0.598 to 1.474, *p* = 0.407) (Figure S5). In addition, previous studies have shown that serum ANG levels are also elevated in women with endometriosis [75], as well as women with severe ovarian hyperstimulation syndrome (OHSS) [76], compared with those in healthy controls.

**Figure 5:**
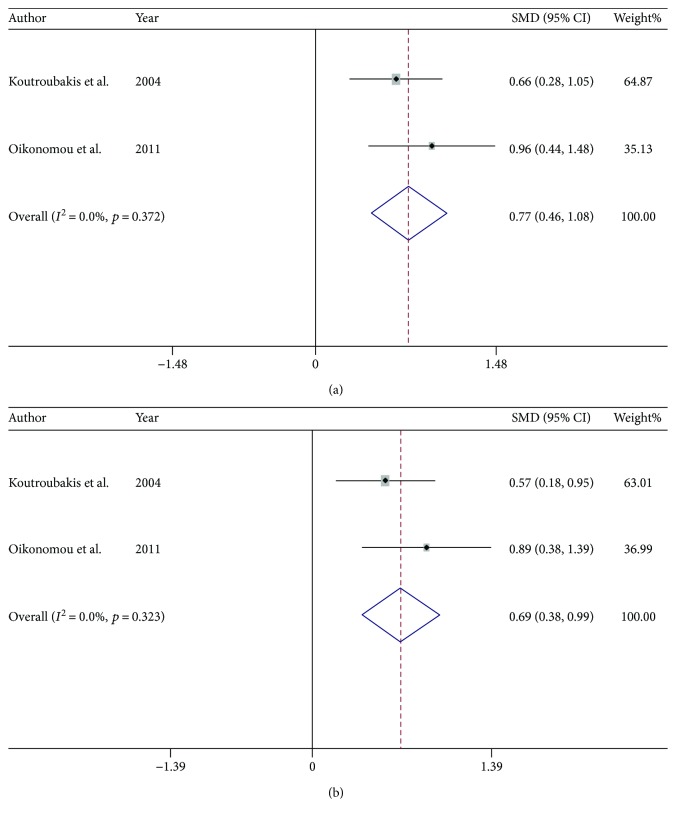
Meta-analysis of studies investigating the relationships between serum ANG levels and IBD. The images of (a) forest plot comparing serum ANG levels in patients with UC with those in healthy controls and (b) forest plot comparing serum ANG levels in patients with CD with those in healthy controls.

Regarding ANG levels in inflammation-related diseases, 2 studies reported that serum ANG levels were increased in patients with inflammatory bowel disease (IBD), that is, patients with ulcerative colitis (UC) and Crohn's disease (CD) [3, 77], compared with those in healthy controls (Figure 5). However, there was no significant difference in serum ANG levels between patients with chronic pancreatitis and healthy volunteers [78], nor was there a difference in serum ANG levels between patients with rheumatoid arthritis and healthy controls [79]. Serum ANG levels were decreased in patients with psoriasis compared with those in healthy controls [80]. In addition, serum ANG levels were significantly increased in patients receiving hemodialysis compared with those in healthy controls [81] and patients with Schnitzler syndrome [82] compared with those in healthy controls.

## 3. Materials and Methods

### 3.1. Search Strategy

This work was conducted and reported in accordance with the standard Meta-analysis of Observational Studies in Epidemiology (MOOSE) guidelines [83]. To find relevant publications, we not only conducted a systematic literature search of PubMed, Web of Science, and Scopus on 1st April 2017 but also manually searched Google Scholar and did not impose publication date restrictions. We used (“angiogenin^∗^”[All Fields]) AND ((“serum”[All Fields] OR “sera”[All Fields]) OR (“plasma”[All Fields]) OR (“circulating”[All Fields])) as search terms in PubMed; TOPIC: (“angiogenin^∗^” OR “RNase-5” OR “ribonuclease 5”) AND TOPIC: (“serum” or “sera” or “plasma” or “circulating”) as search terms in Web of Science; and (TITLE-ABS-KEY ((“angiogenin^∗^” OR “RNase-5” OR “ribonuclease 5”)) AND TITLE-ABS-KEY ((“serum” OR “sera” OR “plasma” OR “circulating”))) as search terms in Scopus. We reviewed the reference sections of the articles identified by the above searches, as well as those of related articles, by hand and then contacted the corresponding authors of the indicated articles to obtain unpublished data and verify the accuracy of the data reported in each study.

### 3.2. Inclusion Criteria

Studies comparing serum ANG levels between patients with specific diseases and healthy controls were included in this study, as were studies evaluating circulating ANG levels in healthy populations, pregnant women, or other demographic populations. Studies included in the analysis were required to present data pertaining to serum ANG concentrations in both patients and healthy controls. In cases in which studies were reported in more than one article, the article reporting data for the largest sample size or the article that was published most recently was included in the analysis.

### 3.3. Exclusion Criteria

The following studies were excluded from the analysis: (a) commentaries or editorials, case reports, and review articles lacking original data; (b) original articles that did not report precise serum ANG levels; (c) studies not involving humans; (d) duplicate reports or studies whose data descriptions were unclear; and (e) studies that reported serum ANG levels were outliers which were approximately 3 orders of magnitude (pg/ml) lower than those of other studies (ng/ml).

Decision regarding candidate article inclusion or exclusion comprised two steps. Pretrained independent reviewers first assessed the titles and abstracts of the candidate articles and then assessed the full texts of these articles to ensure that no relevant articles were excluded from the analysis. Study inclusion or exclusion, as well as study data extraction, was performed by at least two independent reviewers, and any disagreements that arose regarding the extracted data were resolved by achieving consensus.

### 3.4. Quality Assessment

Two investigators independently assessed the methodological quality of the studies included in the analysis using the Newcastle-Ottawa Scale (NOS) [84], a star-based scale that evaluates the quality of individual studies based on group selection, group comparability, and clinical outcome ascertainment. NOS scores range from 0 to 9, and studies whose scores are above 7 were considered good-quality studies.

### 3.5. Data Synthesis and Analysis

Data regarding the following parameters were extracted from each eligible article by two investigators using a standardized form: the first author's name; the year of publication; the country in which the study was performed; the source of the control group; the source of the sample group; the measurement method; the sample size of the patient and control groups; the baseline characteristic of the study participants, including the age and gender of the participants; and the mean and standard deviation of the ANG concentrations in both the patient and the control groups. The median value and interquartile range were transformed into mean and standard deviation, according to the method described by Wan et al. [85].

The standardized mean difference (SMD) was employed to estimate the differences in ANG levels between specific patients and corresponding healthy controls. The SMD and its 95% confidence intervals (CIs) were described by a forest plot. Both the Cochran *Q* statistic and the *I* ^2^ test were used to assess the statistical heterogeneity among the studies [86, 87]. A random-effects model (DerSimonian Laird method) [88] was used in cases in which the results of the *Q* test were significant, with a *p* < 0.05 and/or an *I* ^2^ > 50%. Otherwise, a fixed-effects model (Mantel-Haenszel method) was used. The presence of publication bias was assessed by Begg's tests. Stata version 14.0 software (Stata Corporation, College Station, TX) was used to perform the statistical analysis. The statistical significance threshold was set at 0.05.

## 4. Discussion

This study aims to determine the correlation between serum ANG levels and diseases. Our systematic literature search identified 81 papers that met our inclusion criteria, and most of the studies were eligible for inclusion in the meta-analysis. We found that the average serum ANG level in healthy individuals is 336.14 ± 142.83 ng/ml and that this level remains relatively stable in different populations and regions. Further analysis suggested that serum ANG levels may have the potential of being a marker for cancers and cardiovascular diseases but not for other diseases. Moreover, most of the pooled estimates showed that serum ANG levels were not significantly different between patients and healthy controls and that the heterogeneity between studies was significant with respect to serum ANG levels. It should be noted, however, that the limited number of studies regarding ANG levels in specific diseases, as well as the small sizes of the samples included in those studies, may have resulted in insufficiently high power to detect an association between serum ANG level and a particular disease and thus decreased the robustness of our results.

This systematic review revealed that a considerable amount of heterogeneity was present among different studies, as demonstrated by the high *I^2^* value obtained via meta-analysis. We planned to investigate heterogeneity and explore for publication bias by using funnel plots, but because of the insufficient numbers of studies, this was not possible. We attribute this heterogeneity to several factors, including differences in patient characteristics (such as illness severity, age, and gender), differences in clinical variables (such as treatment dose, treatment timing, or treatment duration), differences in measurement tools, differences in reference standards, or differences in a combination of these factors among studies [89]. Various ELISA kits made by different manufacturers were used to perform serum ANG measurements, which may have resulted in heterogeneity with respect to the measurements [58]. In some cases, serum ANG levels differed by orders of magnitude (from “ng/ml” to “pg/ml”) even when the same ELISA kit (R&D Systems) was used to perform measurements [90, 91]. We recommend that researchers make the following adjustments to overcome the problem of heterogeneity with respect to serum ANG levels: first, researchers should conduct large prospective trials and use consistent criteria to diagnose specific diseases. Second, researchers should use the same ELISA kit to measure serum ANG levels, or they should use specific standards to detect biomarkers using ELISA kits. Researches should also be aware the methods used for serum collection, processing, and storage should meet the standards. Third, researchers should ensure that their studies provide more detailed demographic information and present their results in a uniform manner (means and standard deviations).

Meta-analyses are classified as retrospective studies and do not prove causation. Moreover, causal relationship can be adversely affected by methodological deficiencies in pooled studies. Furthermore, we identified several studies reporting extreme results and outliers that were not eligible for inclusion in the analysis; thus, we were not able to include all the available data regarding serum ANG levels of various populations and diseases in this study.

Our study revealed that serum ANG levels are elevated in cancer, especially CRC, AML, MM, and MDSs. It is not surprising since ANG is an angiogenic and tumorigenic factor. However, elevated serum ANG levels are not associated with all tumor types, perhaps because ANG plays different roles in the pathogenesis of different tumors. Additionally, the results of our analyses may have been affected by the sizes of the samples enrolled in the included studies and by limitations in the numbers of studies regarding the relationships between ANG and specific types of cancer. We were unable to determine an ANG cutoff level because of variations in serum ANG levels among the studies included herein. Moreover, serum ANG levels are also associated with cancer progression. For example, studies have shown that serum ANG levels increase significantly with CRC progression [27, 28]. Serum ANG levels are not elevated in patients with HCC compared with those in healthy controls; however, higher serum ANG levels are significantly associated with HCC histological grades, tumor vascularity, and tumor size [39]. We did not conduct a meta-analysis of the relationship between HCC progression and ANG levels because the studies regarding this relationship did not contain sufficient data. Taken together, these results suggest that serum ANG levels are significantly associated with tumors; however, serum ANG levels vary among different tumor types; therefore, more data are needed to verify the existence of an association between ANG levels and different cancers. We also recommend the performance of large prospective multicenter studies and believe that multidisciplinary teams should collaborate to determine the clinical applicability of serum ANG levels as a biomarker for cancer.

The usefulness of serum ANG as a biomarker for other diseases has also not been established, and many studies investigating the relationships between serum ANG levels and those diseases have obtained contradictory results. Serum ANG levels may be influenced by disease duration, treatment type and duration, sample sources, and measurement methods. For example, we found serum ANG levels may play a role in some types of CVDs. We noted no significant differences in serum ANG levels between patients with CAD and healthy controls. However, the serum ANG levels were significantly elevated in patients with heart failure, the terminal stage of most CVDs, compared with those in healthy controls, which may explain the changes in angiogenic activity noted in affected patients. The processes of angiogenesis appear to be an integral component in the pathophysiology of CVDs, and in heart failure, there are also tissue repairing and inflammatory infiltrating, which can induce the secretion of angiogenic factors including ANG, which may be the reason of raised serum ANG levels.

Our study also observed different roles of ANG between T1DM and T2DM. As we know, diabetes can be broadly subdivided by etiology into T1DM resulting from insulin deficiency and T2DM from resistance to insulin action. The serum ANG levels are changed oppositely between T1DM and T2DM patients with no statistically significant difference. The causes of these issues are that patient age and duration of disease varied widely. From the vascular point of view, diabetes is a paradoxical disease. Excessive and uncontrolled angiogenesis leads to complications such as diabetic retinopathy and nephropathy, while insufficient formation of small blood vessels contributes to impaired wound healing and skin ulcers. ANG is a proangiogenic factor that related to every step of angiogenesis. It is assumed that serum ANG levels are stable in the onset of diabetes but change when accompanied by the two opposite complications. Therefore, further studies in larger cohorts of patients with opposite complications could validate our hypothesis.

A lack of differences in serum ANG concentrations between patients with certain diseases and healthy controls does not indicate that this growth factor has no influence on these diseases. ANG may play a localized role rather than a systemic role in some diseases. In addition to being identified in serum or plasma, ANG has also been identified in normal human fluids, such as amniotic fluid (AF) [92], tumor microenvironment fluid [93], urine [94], and cerebrospinal fluid (CSF) [95]. Three studies investigated CSF ANG levels in patients with ALS [17, 95, 96] (Supplemental Table S2). However, these studies obtained inconsistent results, as one study reported that CSF ANG levels were not significantly different between patients and controls, while the other two studies reported that CSF ANG levels were decreased in patients with ALS compared with those in healthy controls. Three studies measured AF matrix ANG levels upon midtrimester genetic amniocentesis [92, 97, 98] (Supplemental Table S2). All these studies reported elevated AF ANG levels in the mothers of infants who were delivered preterm than those in the mothers of infants who were delivered uneventfully and at term. We did not perform a meta-analysis of the relationship between AF ANG levels and infant gestational age at delivery because we detected between-study heterogeneity with respect to AF ANG levels. Moreover, 6 studies investigated whether ANG could be used as a urinary biomarker for bladder cancer [99–104] (Supplemental Table S2). Eissa et al. reported that ELISA-based quantitative measurements of urinary angiogenic factor levels in voided urine samples were reliable [103], and all the studies included herein that assessed urinary ANG levels in bladder carcinoma reported that ANG levels were significantly elevated in patients with cancer compared with those in healthy controls. However, we could not perform a meta-analysis due to insufficient data regarding this topic. These studies suggested that urinary ANG levels may be useful with respect to diagnosing and monitoring bladder carcinoma; however, larger, prospective studies regarding the relationship between ANG levels and bladder carcinoma are required.

Primary clinical specimens, such as serum specimens, are more representative of the human proteome than other samples. Thus, studies regarding serum ANG level evaluation may be important for optimizing decision making in clinical practice and for understanding the role of ANG in normal physiological and pathophysiological processes. The merits of serum ANG to be used as a biomarker is that, firstly, serum ANG is relatively coincident through different population and region. Secondly, our laboratory experiment has shown that serum ANG is very stable in storage condition. Moreover, the detection method for the serum ANG levels is commercially available. The serum ANG concentrations were significantly elevated in patients who developed colorectal cancer, acute myeloid leukemia, multiple myeloma, myelodysplastic syndromes, and heart failure. Thus, serum ANG might be used as a universal biomarker. Serum ANG level evaluation alone, or with other biomarkers and combined with corresponding clinical symptoms, may be a very useful clinical tool in screening asymptomatic individuals for diseases and would better guide the utilization of resources for expensive medical equipment and decline the frequency of invasive, painful, and expensive examinations. However, before ANG can be implemented in clinical practice, its validity and utility in clinical diseases must be evaluated. This study represents a very early step in the process of implementing ANG in clinical settings.

## 5. Conclusions

Taken together, the findings of our study indicate that serum ANG levels usually remain within a specific range in healthy populations and that they are correlated with various diseases. Serum ANG levels are currently not a clinical diagnostic marker for diseases; however, the significant changes in serum ANG levels that occur in cancers and CVDs indicate that it plays a role in the pathogenesis of these diseases and it may be a potential biomarker for these diseases. Furthermore, the findings of this study may serve as preliminary evidence enabling physicians and researchers to explore its usefulness further in clinical translation studies.

## Supplementary Materials

Supplementary Materials Supplementary Figure 1: forest plot comparing serum ANG levels in males with those in females in a healthy population. All the studies investigating the relationships between ANG levels and gender. Among them, Chung HC also investigated the relationship between ANG levels and breast cancer. Supplementary Figure 2: meta-analysis of studies investigating the relationships between serum ANG levels and different cancers. Images of the (A) forest plot comparing serum ANG levels in patients with CRC with those in healthy controls, (B) forest plot comparing serum ANG levels in patients with AML with those in healthy controls, (C) forest plot comparing serum ANG levels in patients with MM with those in healthy controls, (D) forest plot comparing serum ANG levels in patients with MDS with those in healthy controls, (E) forest plot comparing serum ANG levels in patients with HCC with those in healthy controls, (F) forest plot comparing serum ANG levels in patients with breast cancer with those in healthy controls, (G) forest plot comparing serum ANG levels in patients with NHL with those in healthy controls, and (H) forest plot comparing serum ANG levels in patients with melanoma with those in healthy controls. Supplementary Figure 3: meta-analysis of studies investigating the relationships between serum ANG levels and neurodegenerative diseases. Images of the (A) forest plot comparing serum ANG levels in patients with neurodegenerative diseases with those in healthy controls, (B) forest plot comparing serum ANG levels in patients with AD with those in healthy controls, and (C) forest plot comparing serum ANG levels in patients with ALS with those in healthy controls. Supplementary Figure 4: meta-analysis of studies investigating the relationships between serum ANG levels and diabetes mellitus. Images of the (A) forest plot comparing serum ANG levels in patients with type 1 diabetes with those in healthy controls and (B) forest plot comparing serum ANG levels in patients with type 2 diabetes with those in healthy controls. Supplementary Figure 5: forest plot comparing serum ANG levels in women who developed preeclampsia with those in women who did not.
