# Supplementary material for: The Potential of Angiogenin as a Serum Biomarker for Diseases: Systematic Review and Meta-Analysis
Source: Dis Markers. 2018 Mar 15;2018:1984718. doi: 10.1155/2018/1984718 (PMC5875026; doi:10.1155/2018/1984718)
Supplement: Supplementary Materials — Supplementary Figure 1: forest plot comparing serum ANG levels in males with those in females in a healthy population. All the studies investigating the relationships between ANG levels and gender. Among them, Chung HC also investigated the relationship between ANG levels and breast cancer. Supplementary Figure 2: meta-analysis of studies investigating the relationships between serum ANG levels and different cancers. Images of the (A) forest plot comparing serum ANG levels in patients with CRC with those in healthy controls, (B) forest plot comparing serum ANG levels in patients with AML with those in healthy controls, (C) forest plot comparing serum ANG levels in patients with MM with those in healthy controls, (D) forest plot comparing serum ANG levels in patients with MDS with those in healthy controls, (E) forest plot comparing serum ANG levels in patients with HCC with those in healthy controls, (F) forest plot comparing serum ANG levels in patients with breast cancer with those in healthy controls, (G) forest plot comparing serum ANG levels in patients with NHL with those in healthy controls, and (H) forest plot comparing serum ANG levels in patients with melanoma with those in healthy controls. Supplementary Figure 3: meta-analysis of studies investigating the relationships between serum ANG levels and neurodegenerative diseases. Images of the (A) forest plot comparing serum ANG levels in patients with neurodegenerative diseases with those in healthy controls, (B) forest plot comparing serum ANG levels in patients with AD with those in healthy controls, and (C) forest plot comparing serum ANG levels in patients with ALS with those in healthy controls. Supplementary Figure 4: meta-analysis of studies investigating the relationships between serum ANG levels and diabetes mellitus. Images of the (A) forest plot comparing serum ANG levels in patients with type 1 diabetes with those in healthy controls and (B) forest plot comparing serum ANG levels in patients with [file 1984718.f1.pdf]

Supplementary Table 1. Characteristics and methodological

No.	Diseases	Reference	Year
**Cancers**			
1	Colorectal cancer	Shimoyama S	1999
2		Ramcharan KS	2013
3	Hepatocellular carcinoma	Chao Y	2003
4		Hisai H	2003
5		Chao Y	2013
6	Breast cancer	Chung HC	1998
7		Montero S	1998
8		Duranyildiz D	2009
9		Piven V	2011
10	Pancreatic cancer	Shimoyama S	1996
11	Ovarian cancer	Barton DP	1997
12	Endometrial cancer	Chopra V	1997
13	Urothelial carcinoma	Miyake H	1999
14	Head and neck squamous cell carcinoma	Homer JJ	2002
15	Gastric cancer	Shimoyama S	2003
16	Bladder carcinoma	Zhao H	2005
17	Nasopharyngeal carcinoma	Xia B	2006
18	Acute myeloid leukemia	Brunner B	2002
19		Glenjen N	2002
20		Kim JG	2005
21		Mona AI	2013
22	Multiple myeloma	Alexandrakis MG	2004
23		Kastritis E	2010
24		Akkök CA	2011
25		Terpos E	2012
26		Gkotzamanidou M	2013
27	Relapsed myeloma	Terpos E	2008
18	Myelodysplastic syndromes	Brunner B	2002
28		Alexandrakis MG	2005
29	Non-Hodgkin lymphomas	Passam FH	2008
30		Eldin EN	2012
31	Chronic lymphocytic leukemia	Molica S	2004
20	Chronic myeloid leukemia	Kim JG	2005
20	Acute lymphoblastic leukemia	Kim JG	2005
32	Waldenstrom macroglobulinaemia	Anagnostopoulos A	2007
33	Haematological neoplasias	Negaard HFS	2009
34	Cutaneous T cell lymphoma	Miyagaki T	2012
35	Melanoma	Ugurel S	2001

**36**	**Melanoma**	Tas F	2006
**37**	**Wilms tumor (Nephroblastoma)**	Sköldenberg EG	2001
**Cardiovascular diseases**			
**38**	**Coronary artery disease**	Telo-Montoliu A	2007
**39**		Idriss NK	2010
**40**		Yu P	2016
**38**	**Acute coronary syndrome**	Telo-Montoliu A	2007
**39**		Idriss NK	2010
**41**		Lewicki L	2015
**42**	**Heart failure**	Patel JV	2008
**43**		Jiang H	2014
**40**		Yu P	2016
**44**	**Acute cerebral infarction**	Huang L	2007
**45**	**Cardiovascular diseases**	Bennett PC	2013
**Neurodegenerative Diseases**			
**46**	**Alzheimer's disease**	Kim YN	2012
**47**		Qin W	2015
**48**	**Amyotrophic lateral sclerosis**	Cronin S	2006
**49**		McLaughlin RL	2010
**50**		Van Es MA	2014
**50**	**Parkinson's disease**	Van Es MA	2014
**Diabetes**			
**51**	**Type1 diabetes**	Malamitsi-Puchner A	1998
**52**		Zorena K	2009
**53**		Marek N	2011
**54**		Neubauer-Geryk J	2012
**55**	**Type2 diabetes**	Siebert J	2010
**56**		Ahmed NF	2012
**57**		Gui C	2013
**58**	**Type1&Type2 diabetes**	Maier R	2006
**Other physiological and pathological processes**			
**59**	**Ulcerative colitis**	Koutroubakis IE	2004
**50**		Oikonomou KA	2011
**59**	**Crohn's disease**	Koutroubakis IE	2004
**60**		Oikonomou KA	2011
**61**	**Pre-Eclampsia**	Kolben M	1997
**62**		Reuvekamp A	1999
**63**		Shaarawy M	2005
**64**		Kenny LC	2014
**65**	**Preterm Fetuses**	Lassus P	2003
**66**	**Small-for-gestational-age**	Yamashiro C	2000
**67**	**Pregnancy & hypertension**	Karthikeyan VJ	2012

**68**	**Endometriosis**	Bourlev V	2010
**69**	**Ovarian hyperstimulation syndrome**	Aboulghar MA	1998
**70**	**Chronic pancreatitis**	Shimoyama S	1999
**71**	**Rheumatoid disease**	Bhatia GS	2010
**72**	**Hemodialysis**	Eleftheriadis T	2012
**73**	**Psoriasis**	Miyagaki T	2012
**74**	**Schnitzler Syndrome**	Terpos E	2012
**Healthy population**			
**6**	**Different gender (M/F)**	Chung HC	1998
**75**		Silha JV	2005
**76**		Pantsulaia la	2006
**77**		Aronis K.N.	2011
**78**	**Different age and gender (M/F)**	Malamitsi Puchner A	1999
**79**	**Different age**	Bruserud O	2005
**80**	**Perinatal Period**	Malamitsi Puchner A	1997
**81**	**Menstrual Cycle**	Hayashi K	2000
**45**	**Different race**	Bennett PC	2013

Data are present as median [IQR] or median (range) or mean ± SD;
No., study's number; n, number of the subjects; NOS, Newcastle-Ottawa Scale; Ul
The same No. means from the same study.
a: diabetes complicated with various vascular complications;
b: the unit is “mg/ml”;
c: preterm fetuses ; d: term fetuses born to mothers with diabetes mellitus;
e: Maternal blood; f: Cord blood;
g: nonpregnant controls; h: normotensive pregnant;
All the ELISA means ELISA (R&D Systems), except "i" means ELISA kit (Britis

quality of the included studies

Region	Control			Ca	
	n	Age	ANG (ng/ml)	n	Age
**Epithelial cell group**					
Japan	23	34±9	321.7±59.7	34	62.3±9.2
UK	29	70±6	123.7±94.4	154	73±10
Taiwan	15	NA	341.0±81.3	98	NA
Japan	31	45±14	362.3±84.1	41	63±10
Taiwan	30	NA	343.0±52.4	73	NA
Korea	34	NA	355.0±85.0	34	48(30-66)
Spain	40	NA	206.5±131.5	194	NA
Turkey	75	43(28-69)	244.5±77.8	90	49(27-71)
Malaysia	14	NA	61.6±5.6	8	NA
Germany	16	NA	359.0±59.9	47	NA
UK	11	NA	327.3±95.0	39	NA
USA	20	NA	112.6±38.0	39	NA
Japan	52	NA	337.5±71.4	135	NA
UK	15	47(29-67)	401.4±153.2	30	63(45–80)
Japan	65	30±8	334.1±58.2	123	63.8±12.1
USA	208	62±12	308.0±88.7	209	62.1±12.2
China	30	42(23-62)	292.5±74.2	42	45(24-67)
**Myeloid and lymphoid group**					
Austria	25	NA	255.4±56.6	25	NA
Norway	22	NA	335.0±67.0	22	NA
Korea	6	NA	217.5±77.3	30	42(15-74)
Egypt	25	41.6±11.3	66.0±2.5	60	39.8±13.6
Greece	25	NA	257.4±145.2	65	NA
Greece	35	NA	232.0±56.0	35	NA
Norway	15	46(26-62)	234.4±52.3	15	57(44-67)
Greece	34	66(39-82)	194.0±68.5	174	66(40-94)
Greece	22	NA	165.1±34.3	55	NA
Greece	42	68(43-79)	221.8±62.8	62	67(44-83)
Austria	25	NA	255.4±56.6	65	NA
Greece	15	NA	247.6±151.3	67	54-91
Greece	20	29-58	898.0±119.0	49	18-86
Egypt	20	NA	256.2±89.1	57	NA
Italy	15	NA	598.0±519.2	77	NA
Korea	6	NA	217.5±77.3	14	34(24-62)
Korea	6	NA	217.5±77.3	10	33(16-66)
Greece	30	NA	249.6±62.0	24	NA
Norway	11	23(21-29)	582.0±154.0	93	62(28-89)
Japan	21	40±16	898.0±230.5	36	59.4±13.6
**Others**					
Germany	30	NA	362.1±82.2	125	NA

Turkey	30	NA	324.7±116.7	114	51(18-80)
Sweden	56	3(0-7)	196.0±129.0	14	1.9(0.8–7.5)
Spain	38	67±7	152.6±74.1	44	65±10
UK	25	57±10	203.7±60.3	70	59±10
China	27	NA	279.4±98.8	413	NA
Spain	38	67±7	152.6±74.1	396	67±13
UK	25	57±10	203.7±60.3	24	59±10
Poland	19	60±9	424.9±42.3	33	64.4±3.8
England	112	60±10	316.7±84.1	109	62.8±12.7
China	16	68±8	374.0±53.1	16	76±4
China	26	NA	279.4±98.8	203	NA
China	20	63±9	334.9±93.9	30	67.5±11.2
UK	56	NA	248.0±177.3	108	NA
Korea	18	74.8±6.7	18.1±2.2	20	74.8±6.4
Chinese	262	NA	168.1±40.0	205	NA
Ireland	72	56.7(35-82)	334.6±106.0	79	61.7(31-86)
Ireland	238	NA	467.6±105.4	294	NA
Netherland	231	68(33~95)	401.6±95.9	265	63(23~84)
Netherland	231	68(33~95)	401.6±95.9	163	63(23~84)
Greece	30	14±4	244.7±52.3	40	14.3±3.6
Poland	52	14.9±3.2	212.0±115.2	64	15.1±3.4
Poland	15	55±8	431.7±51.6	20	54.4±5.2
Poland	38	37±6	460(230-708)	57 [a]	39±6.6
Poland	40	NA	472.6±146.3	49 [a]	66.3±10.2
Egypt	20	NA	472.6±45.6	41 [a]	NA
China	36	59±8	4.4±0.85	42	59.2±7.5
Austria	13	NA	1090.0±521.0	13	NA
Greece	42	42	394.6±137.6	78	39
Greece	22	43±10	234.6±131.8	52	49.6±14
Greece	42	42	394.6±137.6	76	39
Greece	23	43±10	234.6±131.8	59	35.9±13.1
German	21	29±5	224.0±79.0	21	30+6
Netherlands Antilles	30	25±7	670.0±252.0	30	27±7
Egypt	20	24.6±0.7	141.0±98.0	71	26.1±0.8
Ireland	5345	29±6	6.9(5.1-9.5) [b]	278	28±6
Finland	42	NA	99.9±4.4	28 [c]	NA
				24 [d]	NA
Japan	47	30(28-34)	242(210–276) [e]	16	26(23-32)
			102(86–115) [f]		
UK	50 [g]	30±8	225(134-331)	38	33±6

	38 [h]	30±6	145(91-230)		
Sweden	21	NA	134.0±73.0	32	NA
Egypt	10	30±5	234.0±91.0	10	30.6±5.2
Japan	14	NA	357.6±45.2	19	NA
UK	57	NA	332.0±62.4	111	30-74
Greece	24	57±9	263.6±66.0	66	61.2±12.5
Japan	21	40±16	898.0±230.5	28	55.8±13.2
France	24	55(30-80)	169.0±33.0	13	55(39-79)
Korea	24/34	NA	282±77/355±85		
Czech Republic	40/61	46.3±2.6/47.8±1.4	483.2±19.8/446.4±13.8		
Georgia	468/468	18-80/18-80	361±104/322±100		
USA	5/5	18-38/18-38	249±56/275±82		
	8/13	NA	110±28/130±43		
	11/17	NA	210±33/250±54		
	17/12	8±3/9±2	220±41/280±45		
Greece	10/12	29±6/30±5	500±126/430±118		
	/11	/NA	/310±99		
	/15	/27±5	/330±77		
	11/13	63±7/63± 9	280±66/470±180		
Norway	14	18	270 (94–400)		
	20	74(68-88)	353 (264–579)		
	10 Maternal	NA	226±50		
	10 Infants d0	NA	119±34		
Greece	30 Infants d1	NA	166±45		
	30 Infants d4	NA	241±53		
	12 Menstrual phase	NA	263±72		
	10 Follicular phase	NA	253±47		
Japan	6 Ovulatory phase	NA	274±49		
	8 Luteal phase	NA	255±56		
	82 South Asian	60±10	240 [135–386]		
UK	84 African Black	66±13	261 [187–367]		
	77 White European	66±11	169 [95–319]		

K: unknow; NA: not available.

h Biotechnology), "j" means ELISA Kit (RayBiotech Inc.) "k" means ELISA kit,

ase ANG (ng/ml)	Source of Control	Method	NOS
411.8±106.3	Volunteer	ELISA	5
309.0±89.1	Staff & Relative	ELISA	8
247.0±22.6	UK	ELISA	6
332.9±143.8	UK	ELISA	5
353.0±58.1	UK	ELISA	8
271.0±106.0	UK	ELISA	7
401.2±167.2	UK	ELISA	6
243.2±69.4	UK	ELISA	8
146.0±107.7	Staff	ELISA	8
566.6±191.9	UK	ELISA [i]	5
643.3±49.6	UK	ELISA	5
273.9±82.6	UK	ELISA	6
435.0±197.5	UK	ELISA	7
469.0±153.3	Hospital	ELISA	6
378.3±95.5	UK	ELISA	5
343.2±108.4	Hospital	ELISA	7
371.4±123.5	Hospital	ELISA	6
305.1±85.5	Blood donors	ELISA	6
458.0±114.7	UK	ELISA	8
306.7±95.0	UK	ELISA	6
212.5±77.1	UK	ELISA [j]	8
658.1±333.5	Staff	ELISA	8
268.6±53.7	UK	ELISA	7
397.4±271.7	Blood donors	ELISA	6
233.5±59.8	UK	ELISA	8
255.6±137.2	UK	ELISA	8
530.0±256.8	UK	ELISA	7
384.8±277.5	Blood donors	ELISA	6
407.5±232.8	UK	ELISA	7
956.0±1735.0	UK	ELISA	6
345.2±154.5	UK	ELISA [k]	6
591.0±338.1	UK	ELISA	7
334.8±98.4	UK	ELISA	6
232.8±61.2	UK	ELISA	6
355.8±248.0	Donors	ELISA	8
710.0±228.0	UK	FACSArray Bioanalyzer	6
1084.0±297.3	UK	ELISA	5
439.0±81.6	UK	ELISA	8

298.3±111.2	UK	ELISA	8
296.0±163.0	UK	ELISA [k]	6
154.4±101.7	Hospital &Relatives	ELISA	8
365.0±190.0	Staff&Relatives&Friends	ELISA	7
309.7±86.7	UK	ELISA	8
258.9±99.1	Hospital &Relatives	ELISA	8
756.0±135.6	Staff&Relatives&Friends	ELISA	7
415.7±42.2	Community	ELISA	8
506.3±319.3	latives&Staff&Commun	ELISA	8
477.0±78.6	UK	ELISA	7
349.9±118.7	UK	ELISA	8
415.1±76.8	Hospital	ELISA	7
257.3±181.1	Relatives&Staff	ELISA	6
15.2±1.0	UK	ELISA [l]	8
195.4±39.0	UK	ELISA [i]	7
396.7±120.9	UK	ELISA	7
438.2±112.2	UK	ELISA	8
425.3±111.3	UK	ELISA	8
399.7±76.4	UK	ELISA	8
353.3±126.5	Staff	ELISA	7
574.3±248.6	UK	ELISA	6
431.7±51.6	UK	ELISA	6
384(190-999)	UK	ELISA	7
392.5±143.2	Hospital	ELISA	8
308.4±38.6	UK	ELISA	7
4.4±1.01	Hospital	Cytokine Array	7
1338.0±648.0	UK	ELISA [i]	/
526.5±224.1	ood donors&visitors&sta	ELISA	8
357.7±126.6	Blood donors	ELISA	8
508.8±228.5	ood donors&visitors&sta	ELISA	8
344.2±120.5	Blood donors	ELISA	8
188.0±62.0	Hospital	ELISA	6
524.0±368.0	Hospital	ELISA	7
547.0±354.3	Hospital	ELISA	8
7.6(5.6-11.0) [b]	Hospital	Luminex Competitive	7
69.3±9.2	Hospital	ELISA	/
96±6<br>199(155–272) [e]<br>90(76–104) [f]	Hospital	ELISA	/
195(145-320)	Hospital	ELISA	/

357.0±84.0	Hospital	ELISA	/
8390.0±6837.0	UK	ELISA	/
352.1±72.5	UK	ELISA	/
335.3±98.5	UK	ELISA	/
499.2±175.7	Staff	ELISA	/
746.4±182.4	UK	ELISA	/
221.0±96.0	UK	ELISA [k]	/

/	UK	ELISA	/
	Hospital	Immunoassays	/
	Community	ELISA	/
	UK	ELISA	/
	Hospital		
	Hospital		
	Hospital		
/	Staff	ELISA	/
	Staff		
	Hospital		
	UK		
/	Community	ELISA	/
/	Hospital	ELISA	/
/	Hospital	ELISA	/
/	Community	ELISA	/

"l" means ELISA (Invitrogen).

Fig. S1

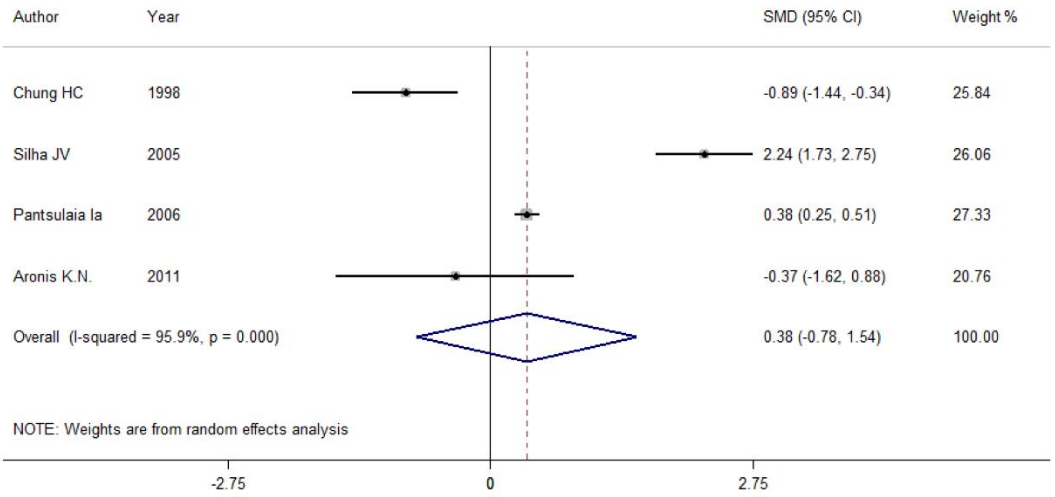

Author
Year
SMD (95% CI)
Weight %
Chung HC
1998
-0.89 (-1.44, -0.34)
25.84
Silha JV
2005
2.24 (1.73, 2.75)
26.06
Pantsulaia Ia
2006
0.38 (0.25, 0.51)
27.33
Aronis K.N.
2011
-0.37 (-1.62, 0.88)
20.76
Overall (I-squared = 95.9%, p = 0.000)
0.38 (-0.78, 1.54)
100.00
NOTE: Weights are from random effects analysis
-2.75
0
2.75

Fig. S2

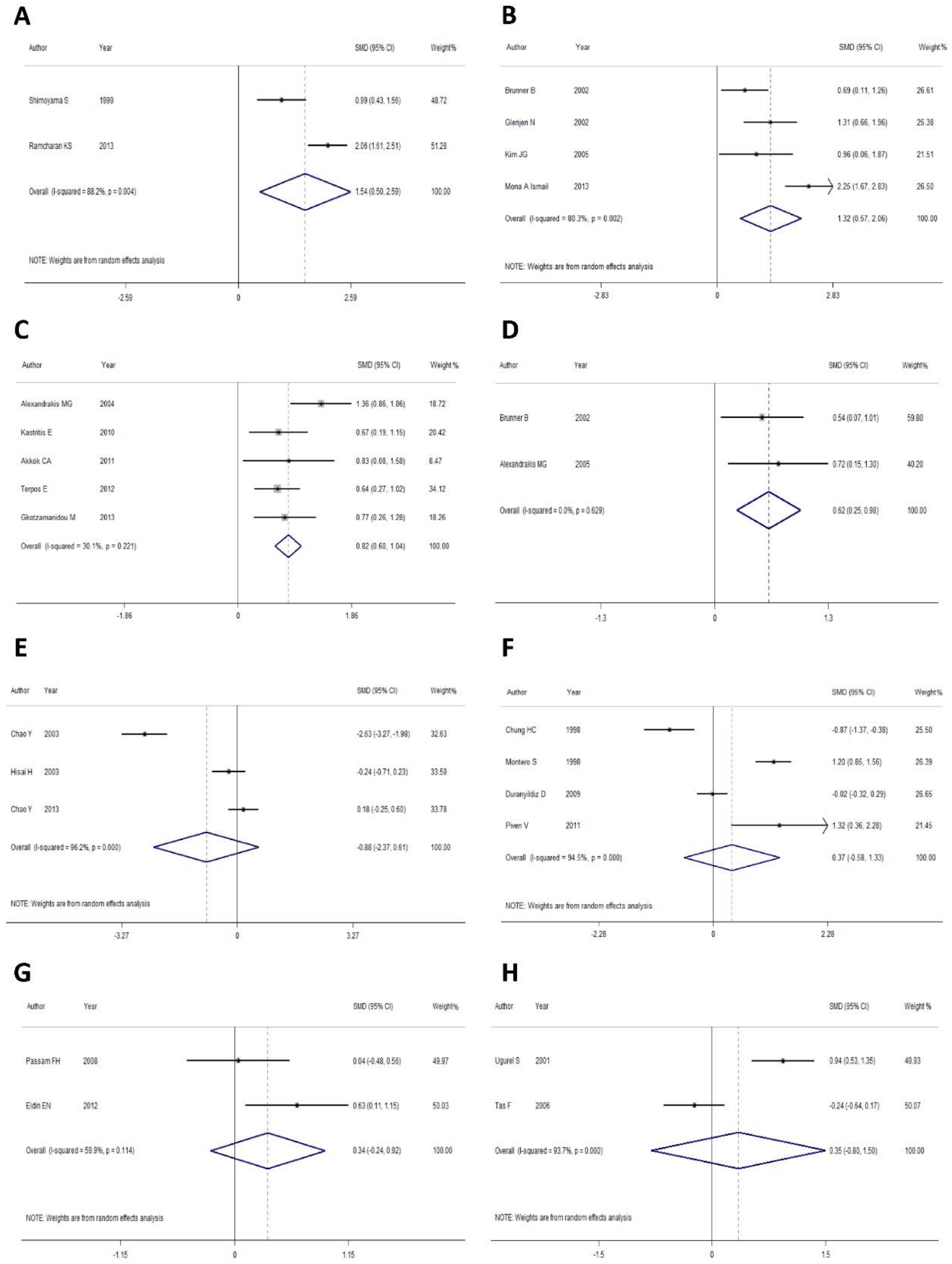
A
Author
Year
SMD (95% CI)
Weight%
Shimoyama S
1999
0.99 (0.43, 1.56)
48.72
Ramcharan KS
2013
2.06 (1.61, 2.51)
51.28
Overall (I-squared = 88.2%, p = 0.004)
1.54 (0.50, 2.59)
100.00
NOTE: Weights are from random effects analysis
-2.59
0
2.59
B
Author
Year
SMD (95% CI)
Weight %
Brunner B
2002
0.69 (0.11, 1.26)
26.61
Glenjen N
2002
1.31 (0.66, 1.96)
25.38
Kim JG
2005
0.96 (0.06, 1.87)
21.51
Mona A Ismail
2013
2.25 (1.67, 2.83)
26.50
Overall (I-squared = 80.3%, p = 0.002)
1.32 (0.57, 2.06)
100.00
NOTE: Weights are from random effects analysis
-2.83
0
2.83
C
Author
Year
SMD (95% CI)
Weight %
Alexandrakis MG
2004
1.36 (0.86, 1.86)
18.72
Kastritis E
2010
0.67 (0.19, 1.15)
20.42
Akkök CA
2011
0.83 (0.08, 1.58)
8.47
Terpos E
2012
0.64 (0.27, 1.02)
34.12
Gkotzamanidou M
2013
0.77 (0.26, 1.28)
18.26
Overall (I-squared = 30.1%, p = 0.221)
0.82 (0.60, 1.04)
100.00
-1.86
0
1.86
D
Author
Year
SMD (95% CI)
Weight%
Brunner B
2002
0.54 (0.07, 1.01)
59.80
Alexandrakis MG
2005
0.72 (0.15, 1.30)
40.20
Overall (I-squared = 0.0%, p = 0.629)
0.62 (0.25, 0.98)
100.00
-1.3
0
1.3
E
Author
Year
SMD (95% CI)
Weight%
Chao Y
2003
-2.63 (-3.27, -1.98)
32.63
Hisai H
2003
-0.24 (-0.71, 0.23)
33.59
Chao Y
2013
0.18 (-0.25, 0.60)
33.78
Overall (I-squared = 96.2%, p = 0.000)
-0.88 (-2.37, 0.61)
100.00
NOTE: Weights are from random effects analysis
-3.27
0
3.27
F
Author
Year
SMD (95% CI)
Weight %
Chung HC
1998
-0.87 (-1.37, -0.38)
25.50
Montero S
1998
1.20 (0.85, 1.56)
26.39
Duranyildiz D
2009
-0.02 (-0.32, 0.29)
26.65
Piwen V
2011
1.32 (0.36, 2.28)
21.45
Overall (I-squared = 94.5%, p = 0.000)
0.37 (-0.58, 1.33)
100.00
NOTE: Weights are from random effects analysis
-2.28
0
2.28
G
Author
Year
SMD (95% CI)
Weight%
Passam FH
2008
0.04 (-0.48, 0.56)
49.97
Eldin EN
2012
0.63 (0.11, 1.15)
50.03
Overall (I-squared = 59.9%, p = 0.114)
0.34 (-0.24, 0.92)
100.00
NOTE: Weights are from random effects analysis
-1.15
0
1.15
H
Author
Year
SMD (95% CI)
Weight %
Ugurel S
2001
0.94 (0.53, 1.35)
49.93
Tas F
2006
-0.24 (-0.64, 0.17)
50.07
Overall (I-squared = 93.7%, p = 0.000)
0.35 (-0.80, 1.50)
100.00
NOTE: Weights are from random effects analysis
-1.5
0
1.5

Fig. S3

A

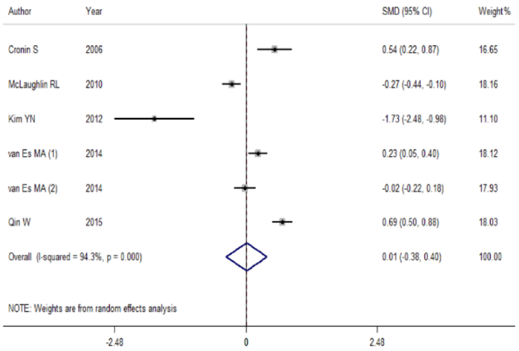

Author
Year
SMD (95% CI)
Weight%
Cronin S
2006
0.54 (0.22, 0.87)
16.65
McLaughlin RL
2010
-0.27 (-0.44, -0.10)
18.16
Kim YN
2012
-1.73 (-2.48, -0.98)
11.10
van Es MA (1)
2014
0.23 (0.05, 0.40)
18.12
van Es MA (2)
2014
-0.02 (-0.22, 0.18)
17.93
Qin W
2015
0.69 (0.50, 0.88)
18.03
Overall (I-squared = 94.3%, p = 0.000)
0.01 (-0.38, 0.40)
100.00
NOTE: Weights are from random effects analysis
-2.48
0
2.48

B

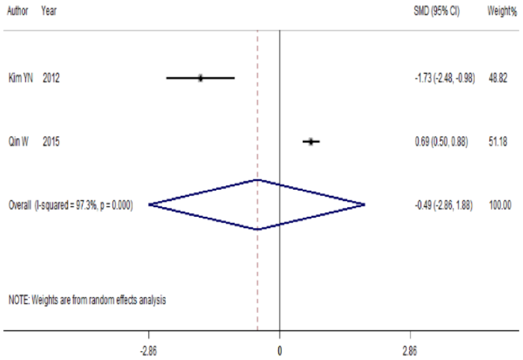

Author
Year
SMD (95% CI)
Weight%
Kim YN
2012
-1.73 (-2.48, -0.98)
48.82
Qin W
2015
0.69 (0.50, 0.88)
51.18
Overall (I-squared = 97.3%, p = 0.000)
-0.49 (-2.86, 1.88)
100.00
NOTE: Weights are from random effects analysis
-2.86
0
2.86

C

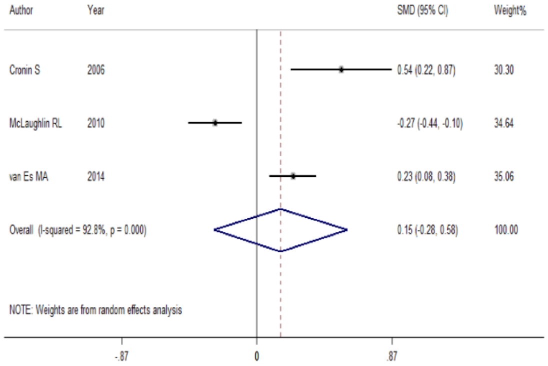

Author
Year
SMD (95% CI)
Weight%
Cronin S
2006
0.54 (0.22, 0.87)
30.30
McLaughlin RL
2010
-0.27 (-0.44, -0.10)
34.64
van Es MA
2014
0.23 (0.08, 0.38)
35.06
Overall (I-squared = 92.8%, p = 0.000)
0.15 (-0.28, 0.58)
100.00
NOTE: Weights are from random effects analysis
-.87
0
.87

Fig. S4

A

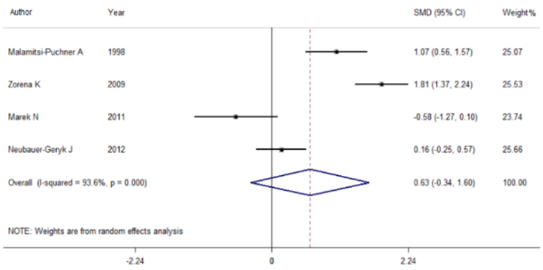
Author
Year
SMD (95% CI)
Weight%
Malamitsi-Puchner A
1998
1.07 (0.56, 1.57)
25.07
Zorena K
2009
1.81 (1.37, 2.24)
25.53
Marek N
2011
-0.58 (-1.27, 0.10)
23.74
Neubauer-Geryk J
2012
0.16 (-0.25, 0.57)
25.66
Overall (I-squared = 93.6%, p = 0.000)
0.63 (-0.34, 1.60)
100.00
NOTE: Weights are from random effects analysis
-2.24
0
2.24

B

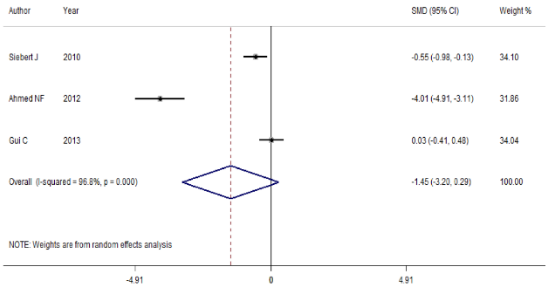
Author
Year
SMD (95% CI)
Weight %
Siebert J
2010
-0.55 (-0.98, -0.13)
34.10
Ahmed NF
2012
-4.01 (-4.91, -3.11)
31.86
Gui C
2013
0.03 (-0.41, 0.48)
34.04
Overall (I-squared = 96.8%, p = 0.000)
-1.45 (-3.20, 0.29)
100.00
NOTE: Weights are from random effects analysis
-4.91
0
4.91

Fig. S5

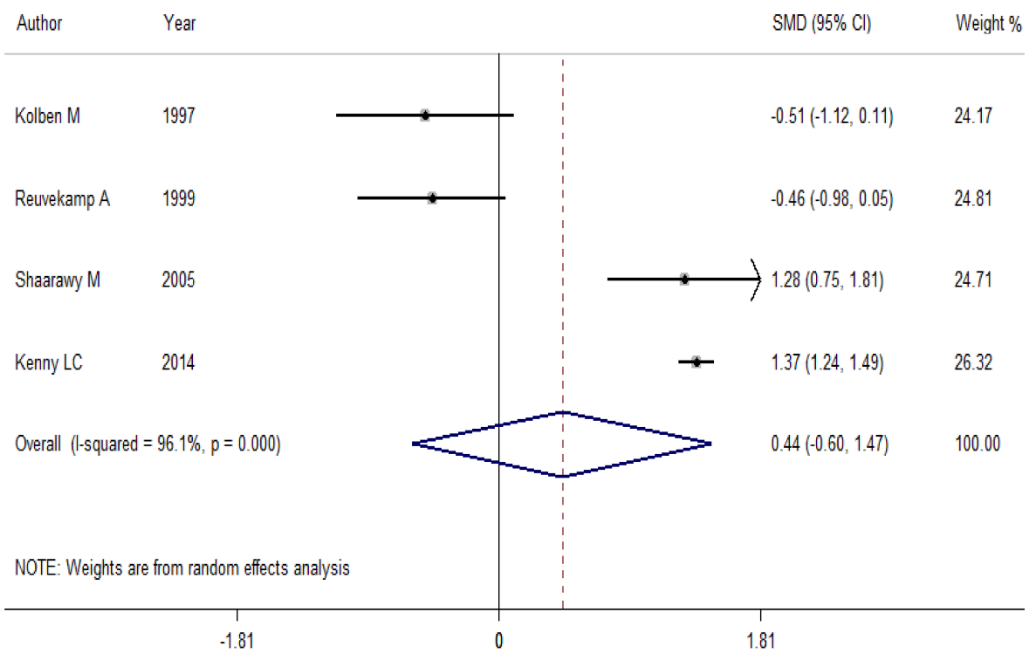
Author
Year
SMD (95% CI)
Weight %
Kolben M
1997
-0.51 (-1.12, 0.11)
24.17
Reuvekamp A
1999
-0.46 (-0.98, 0.05)
24.81
Shaarawy M
2005
1.28 (0.75, 1.81)
24.71
Kenny LC
2014
1.37 (1.24, 1.49)
26.32
Overall (I-squared = 96.1%, p = 0.000)
0.44 (-0.60, 1.47)
100.00
NOTE: Weights are from random effects analysis
-1.81
0
1.81

Supplementary Table 2. Human fluids angiogenin (ANG) levels in patients with those in healthy controls

Reference	Year	Region	Method	Sample size		ANG（ng/mL）	
				Control	Case	Control	Case
**Cerebrospinal fluid ANG levels in patients with amyotrophic lateral sclerosis with those in healthy controls**							
Iłzecka J	2008	Poland	ELISA	20	15	0.328 (0.208–0.45)	0.286 (0.153–0.483)
Moreau C	2009	France	ELISA	40	40	282.5 [244–326]	288.0 [267–307]
McLaughlin RL	2010	Sweden/Ireland/Poland	ELISA	294	238	6.197±1.987	5.582±1.754
**Amniotic fluids ANG levels in maternal with preterm fetuses with those in maternal with full-term fetuses**							
Spong CY	1997	USA	ELISA	33	11	18 (8-43)	30 (14-71)
Yoon BH	2001	Korea	ELISA	95	19	7 (1-22)	11 (5-31)
Madazil R	2003	Turkey	ELISA	45	5[a]	24±9	38±15
					5[b]		49±15
**Urine ANG levels (pg/mL) in patients with bladder carcinoma with those in healthy controls**							
Eissa S	2004	Egypt	ELISA	46	63	116±193.2964[c]	1869.5±3082.829[c]
Eissa S	2009	Egypt	ELISA	110	240	NA	NA
Urquidi V	2012	Florida	ELISA	63	64	44.58 (20.48-696.18)	410.98 (3.28-17944)
Rosser CJ	2013	Orlando/Barcelona	CK400, Cell Sciences	/	102	NA	NA
Shabayek MI	2014	Egypt	ELISA	20	50	27.46 (1233.86)	307.45 (196.16)
Soukup V	2015	Czech Republic	ELISA	49	70	626.4±589.92	1143.11±804.69

Note: NA, not available; [a]: threatened preterm labor; [b]: preterm delivery; [c]: the unit is “pg/mg protein”

All the ELISA means ELISA (R&D Systems);

Data are present as median [IQR] or median (range) or mean ± SD.
